# Identification of Doxorubicin as an Inhibitor of the IRE1α-XBP1 Axis of the Unfolded Protein Response

**DOI:** 10.1038/srep33353

**Published:** 2016-09-16

**Authors:** Dadi Jiang, Connor Lynch, Bruno C. Medeiros, Michaela Liedtke, Rakesh Bam, Arvin B. Tam, Zhifen Yang, Muthuraman Alagappan, Parveen Abidi, Quynh-Thu Le, Amato J. Giaccia, Nicholas C. Denko, Maho Niwa, Albert C. Koong

**Affiliations:** 1 Department of Radiation Oncology, Stanford University School of Medicine, Stanford, CA 94305 USA; 2 Department of Medicine, Stanford University School of Medicine, Stanford, CA 94305 USA; 3 Department of Biological Sciences, University of California, San Diego, CA 92093 USA; 4 Department of Radiation Oncology, The Ohio State University, Columbus, OH 43210 USA

## Abstract

Activation of the IRE1α-XBP1 branch of the unfolded protein response (UPR) has been implicated in multiple types of human cancers, including multiple myeloma (MM). Through an *in silico* drug discovery approach based on protein-compound virtual docking, we identified the anthracycline antibiotic doxorubicin as an *in vitro* and *in vivo* inhibitor of XBP1 activation, a previously unknown activity for this widely utilized cancer chemotherapeutic drug. Through a series of mechanistic and phenotypic studies, we showed that this novel activity of doxorubicin was not due to inhibition of topoisomerase II (Topo II). Consistent with its inhibitory activity on the IRE1α-XBP1 branch of the UPR, doxorubicin displayed more potent cytotoxicity against MM cell lines than other cancer cell lines that have lower basal IRE1α-XBP1 activity. In addition, doxorubicin significantly inhibited XBP1 activation in CD138^+^ tumor cells isolated from MM patients. Our findings suggest that the UPR-modulating activity of doxorubicin may be utilized clinically to target IRE1α–XBP1-dependent tumors such as MM.

Multiple myeloma (MM) is a plasma cell dyscrasia and the second most frequent type of blood cancer in the US, accounting for 13% of hematological malignancies and 1% of all neoplastic diseases^1^. Because of the elevated monoclonal paraprotein secretion, MM cells experience endoplasmic reticulum (ER) stress and engage the unfolded protein response (UPR) as an adaptive strategy to survive and proliferate under these conditions.

Mammalian cells have 3 major UPR signaling branches: inositol-requiring enzyme 1α (IRE1α) - X-Box Binding Protein 1 (XBP1), PKR-like ER kinase (PERK) - activating transcription factor 4 (ATF4), and activating transcription factor 6 (ATF6)^2^. When unfolded proteins accumulate in the ER, IRE1α is activated through autophosphorylation and oligomerization, leading to activation of its endoribonuclease (RNase) activity^3^. Activated IRE1α splices a 26-base-pair intron from *XBP1* mRNA, resulting in a translational frame-shift to encode spliced XBP1 (XBP1s), a potent transcription factor^4^. In addition to its *XBP1* splicing activity, activated IRE1α also preferentially degrades ER-associated mRNAs through cleavage at both stem-loop sites and non-stem-loop sites, a process referred to as regulated IRE1-dependent decay (RIDD). This process is thought to reduce the folding load of nascent proteins in the ER and maintain ER homeostasis^5^,^6^,^7^,^8^. XBP1 is highly expressed in human MM cells^9^. Inhibition of XBP1 expression by siRNA sensitizes myeloma cells to stress-induced apoptosis^10^ and XBP1 overexpression recapitulates MM pathogenesis in a mouse model^11^. The results from these studies are consistent with clinical reports showing that XBP1s expression is associated with poor MM patient survival^12^. Despite recent advances in therapy, MM still remains incurable and overall survival following treatment is 3–7 years^1^. Therapeutic strategies based upon modulating the UPR are effective in cancer treatment and FDA approval of the proteasome inhibitor bortezomib for treating MM is an example of a cancer therapy targeting the UPR^13^. Papendreou *et al.* first reported the feasibility of inhibiting the IRE1α-XBP1 pathway with a small molecule inhibitor^14^. Since then, other IRE1α-XBP1 inhibitors and their associated cytotoxicity on MM cells have been reported^15^,^16^,^17^.

In this study, we applied an *in silico* approach to identify small molecules that structurally fit into the endoribonuclease domain of IRE1α based on its crystal structure^18^. After screening a small ligand library of clinically-utilized drugs, we identified doxorubicin, an anthracycline antibiotic, as a novel inhibitor of the IRE1α-XBP1 pathway. Our findings thus reveal a previously unknown activity of doxorubicin and support a preclinical rationale for applying this drug in treating pathological conditions where elevated IRE1α-XBP1 signaling is implicated.

## Results

**Figure 1:**
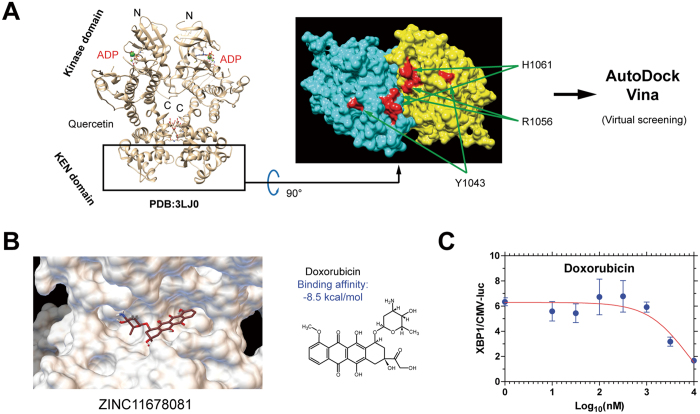
Identification of doxorubicin as an inhibitor of the IRE1α-XBP1 axis. (**A**) Left: Crystal structure of the cytoplasmic portion (kinase domain and kinase extension nuclease, KEN domain) of yeast IRE1α dimer (PDB#:3LJ0), with ADP and quercetin (a flavonol that activates IRE1α endoribonuclease activity) bound. Right: Bottom view of the IRE1α dimer where the endoribonuclease domain and RNA substrate recognition site are located. Individual monomers are colored in blue and yellow. The 3 residues (H1061, R1056 and Y1043) critical for endoribonuclease activity are labeled in red. (**B**) Docking (left) and binding affinity (right) of doxorubicin to the endoribonuclease domain of IRE1α. The ZINC database ID# of doxorubicin is shown on the left. (**C**) Log [inhibitor] response curve of doxorubicin defined as the ratio of luciferase activity measured from HT1080-XBP1-luc cells and that from the HT1080-CMV-luc (a control for non-specific toxicity) cells treated with 300 nM thapsigargin and varying doses of doxorubicin for 12 hours.

The reported crystal structure of the IRE1α protein provided a unique opportunity for structure-based drug identification based upon disrupting the interaction between the endoribonuclease domain and its mRNA substrate^18^. AutoDock (AD) Vina is a newly-developed computing program for molecular docking with improved performance and accuracy compared to predecessors^19^. Applying AD Vina with a grid box encompassing the endoribonuclease domain of the IRE1α dimer, we performed a virtual screen on a library of clinically-utilized drugs (Fig. 1A). Interestingly, the anthracycline antibiotic doxorubicin was identified to have a high predicted binding affinity (−8.5 kcal/mol), although this drug has not been known to influence the IRE1α-XBP1 pathway previously (Fig. 1B). We confirmed its inhibition on IRE1α-induced *XBP1* splicing using an XBP1-luciferase reporter cell line (HT1080-XBP1-luc) normalized to the control HT1080-CMV-luc reporter cell line^20^ (Fig. 1C).

**Figure 2:**
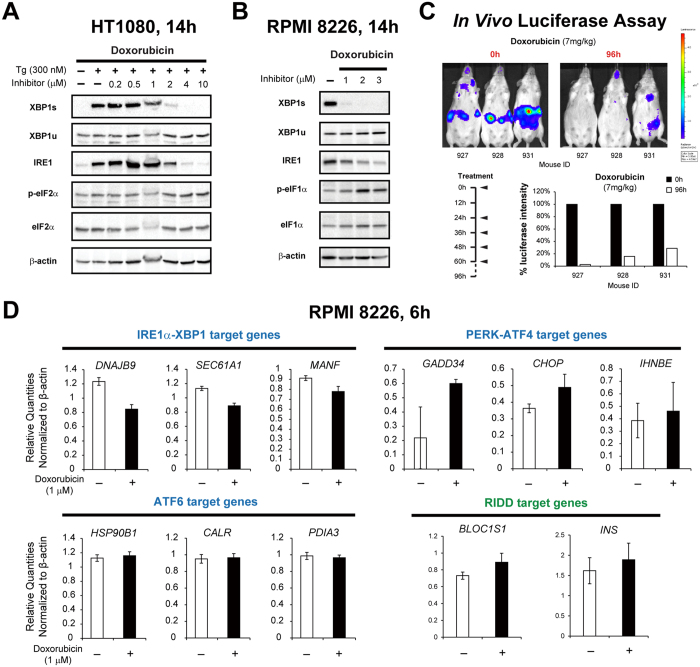
*In vitro* and *in vivo* inhibition of the IRE1α-XBP1 pathway by doxorubicin. (**A**) Western blot analysis of UPR signaling proteins in HT1080 cells treated with 300 nM thapsigargin together with varying doses of doxorubicin for 14 hours. XBP1s denotes spliced XBP1 and XBP1u denotes unspliced XBP1. β-actin was used as a loading control. (**B**) Western blot analysis of UPR signaling proteins in RPMI 8226 cells treated with varying doses of doxorubicin for 14 hours. β-actin was used as a loading control. (**C**) Top: 0-hour and 96-hour bioluminescent imaging of XBP1-luc transgenic mice injected with 7 mg/kg doxorubicin intraperitoneally. Color scheme shows the radiance level. Lower left panel: Treatment schedule with arrows indicating the time points for each treatment. Lower right panel: histograms summarize the bioluminescent signals normalized to 0 hour, which is set to 100%. (**D**) Expression levels of representative target genes of different UPR branches as well as 2 RIDD target genes in RPMI 8226 cells after no treatment or 1 μM doxorubicin treatment for 6 hours analyzed by qRT-PCR. Results represent average quantities of technical triplicates normalized to β-actin ± c.v. (coefficient of variation).

Next, we treated human HT1080 fibrosarcoma cells with 300 nM thapsigargin (Tg, an inhibitor of ER Ca^2+^ ATPase that causes ER stress) and assessed the effect of doxorubicin on the expression of different UPR signaling proteins (Fig. 2A). Consistent with the results of the luciferase reporter assay, doxorubicin completely blocked Tg-induced XBP1s expression at ≥2 μM. Meanwhile, the total level of IRE1α protein decreased, with kinetics matching the inhibition of XBP1s, consistent with a previous report^21^. In contrast, Tg-induced phosphorylation of eIF2α, a marker of activation of the PERK-ATF4 branch of UPR, was not significantly affected by doxorubicin treatment. To demonstrate that doxorubicin directly inhibits IRE1α RNase activity, we performed *in vitro* nuclease assays to test doxorubicin inhibition on two substrates, XBP1 and BLOC1S1 (BLOS1), representing *XBP1* splicing and RIDD^8^ activities, respectively. We found that doxorubicin inhibited IRE1α activity *in vitro* with an IC_50_ of ~20 μM for both XBP1 and RIDD (Supplementary Fig. S1). These data indicate that in a cell-free assay, doxorubicin directly inhibits IRE1α in a concentration-dependent manner. To ascertain whether this inhibitory effect applies to the high endogenous IRE1α-XBP1 activity in MM cells, we examined the expression of the same set of UPR signaling proteins under basal conditions in a panel of MM cells exposed to doxorubicin (Fig. 2B and Supplementary Fig. S2). We found that doxorubicin completely inhibited XBP1s at ≥2 μM. Interestingly, it concomitantly increased eIF2α phosphorylation in some of these cells, suggesting a feedback modulation of the PERK-ATF4 pathway. To compare the effect of doxorubicin to that of another inhibitor of IRE1α RNase activity^14^,^22^,^23^, we treated HT1080 and RPMI 8226 cells with doxorubicin or STF-083010 under thapsigargin-induced UPR. Compared to STF-083010, doxorubicin showed greater potency in inhibiting XBP1s expression (Supplementary Fig. S3A,B) and caused greater cytotoxicity in multiple MM cell lines (Supplementary Fig. S3C). To extend our observations to an *in vivo* setting, we utilized transgenic mice expressing the XBP1-luciferase reporter in which activation of XBP1s can be detected through bioluminescence imaging^20^. Similar to its *in vitro* activity, intraperitoneally-administered doxorubicin also inhibited *in vivo XBP1* splicing in the normal tissues of these mice (Fig. 2C).

To clarify the effect of doxorubicin on the UPR, we analyzed the expression of representative target genes of the three UPR branches as well as RIDD activity, in RPMI 8226 cells after doxorubicin treatment (Fig. 2D). Consistent with the protein expression analysis, doxorubicin significantly repressed the expression of target genes of the IRE1α-XBP1 branch, while inducing those of the PERK-ATF4 branch. In contrast, transcriptional activity of the ATF6 branch was not affected. Interestingly, the RIDD activity of IRE1α was not significantly inhibited by doxorubicin, as shown by the expression levels of two RIDD target genes (*BLOC1S1*^24^ and *INS*^7^) in this setting.

**Figure 3:**
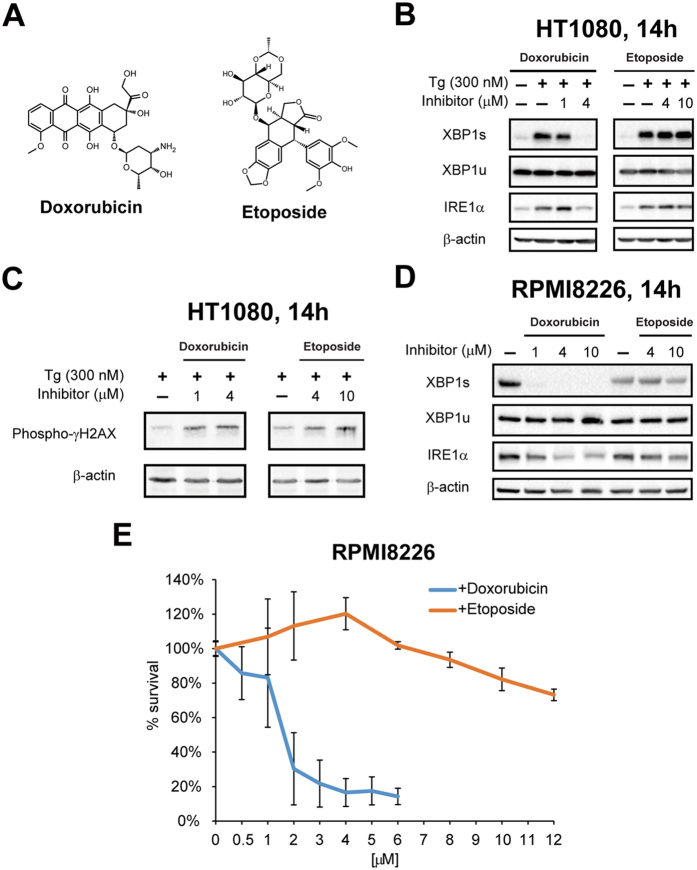
The inhibitory effect on *XBP1* splicing of doxorubicin is independent of Topo II inhibition. (**A**) Chemical structures of doxorubicin and etoposide. (**B**) Western blot analysis of the IRE1α-XBP1 branch in HT1080 cells treated with 300 nM thapsigargin together with varying doses of doxorubicin or etoposide for 14 hours. β-actin was used as a loading control. (**C**) Extent of DNA damage as measured by Western blot analysis of phospho-γH2AX in HT1080 cells treated with 300 nM thapsigargin together with varying doses of doxorubicin or etoposide for 14 hours. β-actin was used as a loading control. (**D**) Western blot analysis of the IRE1α-XBP1 branch in RPMI 8226 cells treated with varying doses of doxorubicin and etoposide for 14 hours. β-actin was used as a loading control. (**E**) XTT cell viability assay for RPMI 8226 multiple myeloma cells treated with varying doses of doxorubicin or etoposide for 24 hours. Values represent % viable cells normalized to no treatment (set to 100%) ± SD.

The most well-established mechanism of action of doxorubicin as a chemotherapeutic drug is through inhibition of topoisomerase II (Topo II) hence blocking DNA replication and causing DNA cleavage^25^. We first determined whether inhibition of IRE1α-XBP1 by doxorubicin could also be due to Topo II inhibition. To address this hypothesis, we compared the effects of doxorubicin and etoposide, another well-known Topo II inhibitor that shares a similar DNA-damaging and cytotoxic profile as doxorubicin^26^, on UPR activation (Fig. 3A). In HT1080 cells treated with Tg, doxorubicin showed potent inhibition of *XBP1* splicing below 4 μM while etoposide had no effect even at 10 μM (Fig. 3B). This result was not due to differences in their potency to induce DNA damage, resulting from the Topo II inhibitory activity, as both drugs equally increased the level of phospho-γH2AX, a marker of DNA damage, at this dose range (Fig. 3C). Similarly, in RPMI 8226 cells, doxorubicin completely blocked endogenous *XBP1* splicing at 1–10 μM, while etoposide had no effect on *XBP1* splicing in the same dose range (Fig. 3D). Furthermore, RPMI 8226 cells were relatively resistant to etoposide treatment, in contrast to the potent killing by doxorubicin at ≥2 μM (Fig. 3E). These results suggest that Topo II inhibition only partly contributes to the mechanism of cell death in RPMI 8226 cells and that inhibition of XBP1s is an important, previously unrecognized activity of doxorubicin. To further support this conclusion, we inhibited the expression of either *TOP2A* or *TOP2B*, which encodes one of the two Topo II isoforms, in RPMI 8226 cells using an siRNA approach (Supplementary Fig. S4A). Knock-down of the expression of either gene did not significantly change the sensitivity of RPMI 8226 cells to doxorubicin treatment (Supplementary Fig. S4B), suggesting that the cytotoxicity of doxorubicin on MM cells is largely independent of topoisomerase II.

**Figure 4:**
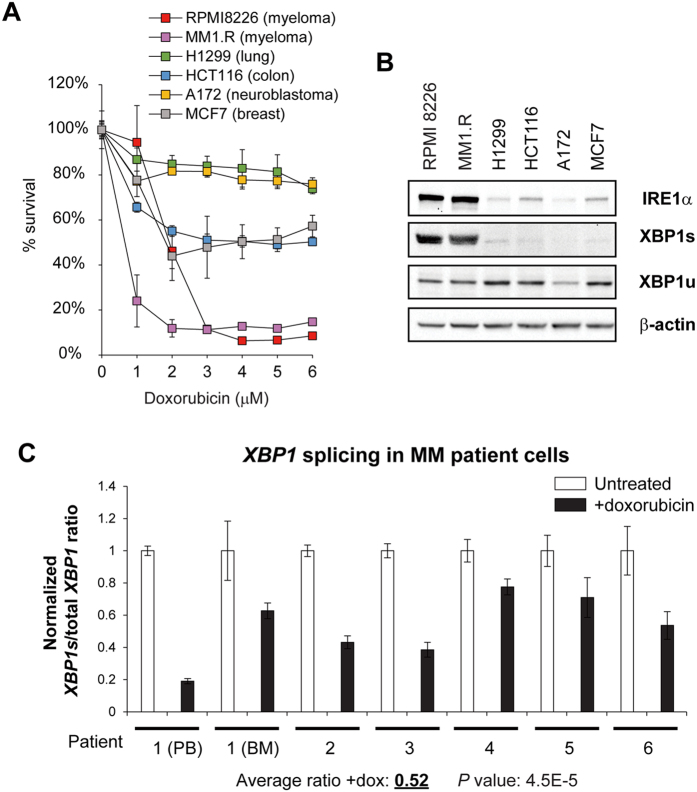
Doxorubicin preferentially induces cytotoxiciy in MM cell lines and inhibits *XBP1* splicing in MM patient cells. (**A**) XTT cell viability assay of RPMI 8226 and MM1.R MM cells and 4 other cancer cell lines (H1299 non-small cell lung carcinoma, HCT116 colorectal carcinoma, A172 glioblastoma and MCF7 mammary adenocarcinoma) treated with varying doses of doxorubicin for 24 hours. Values represent % viable cells normalized to no treatment (set to 100%) ± SD. (**B**) Western blot analysis of IRE1α and XBP1 proteins for the cell lines used in (**A**). (**C**) Doxorubicin inhibited endogenous *XBP1* mRNA splicing in CD138^+^ cells freshly isolated from MM patients undergoing active therapy. CD138^+^ cells isolated from 6 MM patients were treated with 1 μM doxorubicin for 6 hours then total RNA was extracted and used for qRT-PCR analysis with primers specific for either spliced or total *XBP1*. Results represent average quantities of technical triplicates of spliced *XBP1 (XBP1s*) normalized to total *XBP1* ± c.v. Results of CD138^+^ cells from both peripheral blood (PB) and bone marrow (BM) of patient 1 are shown.

To confirm that the specific UPR-modulating activity of doxorubicin can be translated into increased tumor cell killing in MM, we also performed XTT viability assays on RPMI 8226, MM1.R (dexamethasone-resistant), U266 and NCI-H929 human MM cells treated with varying doses of doxorubicin. Doxorubicin showed significant cytotoxicity on these cell lines, with NCI-H929 and MM1.R cells being more sensitive to the treatment than the other two cell lines (Fig. 4A and Supplementary Fig. S3C). The cytotoxic effect is mainly due to the induction of apoptosis, and an increase in G_2_/M arrest was also observed (Supplementary Figs S5 and S6). In contrast, other cancer cell lines, including those that originated from lung (H1299), colon (HCT116), breast (MCF7) and CNS (A172), showed very low basal IRE1α expression and almost undetectable XBP1s expression compared to the MM cells (Fig. 4B). These cells displayed much less sensitivity to doxorubicin compared to MM cells in the same dose range (Fig. 4A). This observation suggests that doxorubicin preferentially induces cytotoxicity in tumor cells with higher basal IRE1α-XBP1s expression. To ascertain the clinical relevance of this finding, we further examined whether this drug also blocked *XBP1* splicing in MM patients. We isolated CD138^+^ MM cells from patients with active disease and found that *XBP1* splicing was significantly inhibited by doxorubicin at 1 μM (Fig. 4C), which is within the previously reported cytotoxic dose range of this drug against human leukemic cells^27^. Consistent with the analysis in RPMI 8226 cells (Fig. 2D), doxorubicin treatment also induced the expression of target genes of the PERK-ATF4 branch in CD138^+^ MM patient cells (Supplementary Fig. S7).

## Discussion

Through an *in silico* approach, our study revealed a previously unrecognized activity of a clinically-utilized drug in inhibiting the IRE1α-XBP1 axis of the UPR. Although the established mechanism of action of doxorubicin is through DNA/RNA intercalation and Topo II inhibition, this study indicates that it can also block *XBP1* splicing as a separate mechanism for cytotoxicity in MM cells, suggesting additional clinical indications for this chemotherapeutic agent. In support of our finding, previous studies of anthracyclines for use in MM^28^ and combinations of bortezomib and pegylated liposomal doxorubicin (PegLD) treatment in MM patients have demonstrated anti-tumor activity^29^. Interestingly, doxorubicin is known to cause cardiotoxicity, in which ER stress is also recognized to play a role^30^. Furthermore, we also observed a concomitant decrease in total IRE1α protein level upon doxorubicin treatment, in the presence of either chemically-induced (HT1080 cells with thapsigargin treatment) or high level of endogenous ER stress (MM cells) (Fig. 2, Supplementary Figs S2 and S3). This is in contrast to results from earlier studies, in which genetic deficiency of *XBP1*^31^ or pharmacological inhibition of *XBP1*^17^,^23^ splicing led to increased expression of IRE1α protein in B cells. This difference may be specific to the cell type (myeloma versus B cells) or stress context (acute or high level of ER stress versus physiological level of ER stress). In summary, our study provides a molecular mechanism and preclinical rationale for using doxorubicin against MM, as well as other types of cancers in which IRE1α-XBP1 signaling plays a prominent role in their pathogenesis. And finally, this study demonstrates the feasibility of utilizing similar *in silico* screening techniques for drug discovery.

## Methods

### Virtual screening

The crystal structure of yeast IRE1α (PDB ID: 3LJ0) was obtained from RCSB Protein Data Bank. The grid box for AutoDock Vina (AD Vina) was generated using PyRx with these parameters: center_x = 92.9680/y = −59.0127/z = 23.9068, size_x = 17.2531/y = 28.7066/z = 32.5466. The virtual screen was performed using AD Vina (version 1.1.2, The Scripps Research Institute). The compound library screen was prepared using the ZINC database (http://zinc.docking.org/pdbqt/), which contains 3180 forms of clinically-utilized compounds in pdbqt file format. The same approach has been tested on other protein targets and the results are being validated separately.

### Cell culture, reporter assay, cell-free IRE1α RNase assay, Western blotting, qRT-PCR, cell viability assay and bioluminescent imaging

RPMI 8226 and MM1.R multiple myeloma cells were maintained in RPMI 1640 medium supplemented with 10% FCS. HT1080, H1299, HCT116, A172 and MCF7 cells were maintained in DMEM medium supplemented with 10% FCS. Standard tissue culture conditions (20% O_2_, 5% CO_2_ and 37 °C) were applied to all the cell lines. Doxorubicin HCl and etoposide were obtained from APP Pharmaceuticals (Schaumburg, IL) and Sigma-Aldrich (St. Louis, MO), respectively. XBP1-luc reporter assay was described previously^20^. Standard assay conditions were used for Western blotting. Antibodies used include: anti-XBP1s (1:1000, BioLegend, San Diego, CA), anti-XBP1 (1:1000, Abcam, Cambridge, MA), anti-IRE1α, anti-eIF2α/phospho-eIF2 (1:1000, Cell Signaling, Danvers, MA) and anti-β actin (Santa Cruz Biotech, Santa Cruz, CA). qRT-PCR was performed using SYBR green and a 7900HT Fast Real-Time PCR machine (Applied Biosystems). qPCR primers used: *DNAJB9* Forward: TGGCCTCAAAAAGCTACTATGATATCTT Reverse: CCAACTTGTGAAAGGCCTTCTT; *SEC61A1* Forward: GGCCACACGCACAGACAAG Reverse: AGATTCATGAGGTTGGGAAGATTC; *MANF* Forward: AGCCTGGAGCTTTCCTGATG Reverse: GTCCTAGAGTACACCAGCAACAGAAG; *GADD34* Forward: TCCCAGTTGTTGATCTTATGCAA Reverse: AAGTGCCGTGGCGACAAG; *CHOP* Forward: GAGAACCAGGAAACGGAAACAG Reverse: TCTCCTTCATGCGCTGCTTT; *INHBE* Forward: GGCAGCCCAGGCATTG Reverse: CCAAGGATTGTTGGCTTTGAG; *HSP90B1* Forward: TTCTTTTTGGGAGAGACTTGTTTTG Reverse: TGACCCATAATCCCACATTTTACA; *CALR* Forward: AGTCCGGCTCCTTGGAAGA Reverse: TCTTCCGGTTTTGAAGCATCA; *PDIA3* Forward: CCAATGATGTGCCTTCTCCAT Reverse: TCACGGCCACCTTCATATTTC; *BLOC1S1* Forward: AGAACTGGGCTCGGAGCAT Reverse: AGCTGCCCTTTGTAGACATAT *INS* Forward: GAAGCTCTCTACCTAGTGTGCGG Reverse: CTCCAGGGCCAAGGGCT; *Total XBP1*: Forward: GTGAAGGAAGAACCTGTAGAAGATGA Reverse: TTTGGGCAGTGGCTGGAT; *XBP1s*: Forward: GCTGAGTCCGCAGCAGGT Reverse: TTGAAGAACATGACTGGGTCCA; *ACTB* Forward: ATCCGCCGCCCGTCCACA Reverse: ACCATCACGCCCTGGTGCCT. For cell viability assay, 2 × 10^4^ cells per well were plated into 96-well plates and treatment started 0–12 hours after plating. After 24 hours, XTT reagent (ATCC, Manassas, VA) was added to the wells then incubated for 2 hours. Cell viability was calculated with the formula: Absorbance = A_475nm_(Test) − A_475nm_(Blank) − A_660nm_(Test) using a BioTek Synergy H1 plate reader (BioTek, Winooski, VT). *In vivo* luciferase activity was measured noninvasively using the IVIS imaging system (PerkinElmer, Waltham, MA) as described previously^20^. *In vivo* bioluminescent signal was quantified by taking the average photon count per second per square centimeter. All experimental procedures were approved and conducted in accordance with the guidelines of the Administrative Panel on Laboratory Animal Care (APLAC) of Stanford University (Protocol Number: 9981).

### Human specimen isolation

Peripheral blood and/or bone marrow aspiration samples were obtained from MM patients after obtaining informed consent with approval from the Institutional Review Board of Stanford University. All methods were performed in accordance with the relevant guidelines and regulations. CD138^+^ plasma cells were selected using Lymphoprep (StemCell Technologies, Vancouver) for mononuclear cell isolation followed by positive magnetic bead selection (StemCell Technologies, Vancouver) and cultured in RPMI 1640 medium plus 10% FCS with and without doxorubicin for 6 hours. Total RNA was extracted using Trizol Reagent (Life Technologies).

## Additional Information

**How to cite this article**: Jiang, D. *et al.* Identification of Doxorubicin as an Inhibitor of the IRE1α-XBP1 Axis of the Unfolded Protein Response. *Sci. Rep.* **6**, 33353; doi: 10.1038/srep33353 (2016).

## Supplementary Material

Supplementary Information

## References

[b1] Raab M. S., Podar K., Breitkreutz I., Richardson P. G. & Anderson K. C. Multiple myeloma. Lancet 374, 324–339 (2009). 19541364 10.1016/S0140-6736(09)60221-X

[b2] Walter P. & Ron D. The unfolded protein response: from stress pathway to homeostatic regulation. Science 334, 1081–1086 (2011). 22116877 10.1126/science.1209038

[b3] Korennykh A. V. *et al.* The unfolded protein response signals through high-order assembly of Ire1. Nature 457, 687–693 (2009). 19079236 10.1038/nature07661 PMC2846394

[b4] Yoshida H., Matsui T., Yamamoto A., Okada T. & Mori K. XBP1 mRNA is induced by ATF6 and spliced by IRE1 in response to ER stress to produce a highly active transcription factor. Cell 107, 881–891 (2001). 11779464 10.1016/s0092-8674(01)00611-0

[b5] Hollien J. & Weissman J. S. Decay of endoplasmic reticulum-localized mRNAs during the unfolded protein response. Science 313, 104–107 (2006). 16825573 10.1126/science.1129631

[b6] Hollien J. *et al.* Regulated Ire1-dependent decay of messenger RNAs in mammalian cells. J Cell Biol 186, 323–331 (2009). 19651891 10.1083/jcb.200903014 PMC2728407

[b7] Han D. *et al.* IRE1alpha kinase activation modes control alternate endoribonuclease outputs to determine divergent cell fates. Cell 138, 562–575 (2009). 19665977 10.1016/j.cell.2009.07.017 PMC2762408

[b8] Tam A. B., Koong A. C. & Niwa M. Ire1 has distinct catalytic mechanisms for XBP1/HAC1 splicing and RIDD. Cell reports 9, 850–858 (2014). 25437541 10.1016/j.celrep.2014.09.016 PMC4486022

[b9] Munshi N. C. *et al.* Identification of genes modulated in multiple myeloma using genetically identical twin samples. Blood 103, 1799–1806 (2004). 12969976 10.1182/blood-2003-02-0402

[b10] Lee A. H., Iwakoshi N. N., Anderson K. C. & Glimcher L. H. Proteasome inhibitors disrupt the unfolded protein response in myeloma cells. Proc Natl Acad Sci USA 100, 9946–9951 (2003). 12902539 10.1073/pnas.1334037100 PMC187896

[b11] Carrasco D. R. *et al.* The differentiation and stress response factor XBP-1 drives multiple myeloma pathogenesis. Cancer Cell 11, 349–360 (2007). 17418411 10.1016/j.ccr.2007.02.015 PMC1885943

[b12] Bagratuni T. *et al.* XBP1s levels are implicated in the biology and outcome of myeloma mediating different clinical outcomes to thalidomide-based treatments. Blood 116, 250–253 (2010). 20421453 10.1182/blood-2010-01-263236

[b13] Field-Smith A., Morgan G. J. & Davies F. E. Bortezomib (Velcadetrade mark) in the Treatment of Multiple Myeloma. Ther Clin Risk Manag 2, 271–279 (2006). 18360602 10.2147/tcrm.2006.2.3.271 PMC1936263

[b14] Papandreou I. *et al.* Identification of an Ire1alpha endonuclease specific inhibitor with cytotoxic activity against human multiple myeloma. Blood 117, 1311–1314 (2011). 21081713 10.1182/blood-2010-08-303099 PMC3056474

[b15] Hetz C., Chevet E. & Harding H. P. Targeting the unfolded protein response in disease. Nat Rev Drug Discov 12, 703–719 (2013). 23989796 10.1038/nrd3976

[b16] Jiang D., Niwa M. & Koong A. C. Targeting the IRE1alpha-XBP1 branch of the unfolded protein response in human diseases. Seminars in cancer biology (2015). 10.1016/j.semcancer.2015.04.010 PMC4523453 25986851

[b17] Tang C. H. *et al.* Inhibition of ER stress-associated IRE-1/XBP-1 pathway reduces leukemic cell survival. The Journal of clinical investigation 124, 2585–2598 (2014). 24812669 10.1172/JCI73448 PMC4038575

[b18] Wiseman R. L. *et al.* Flavonol activation defines an unanticipated ligand-binding site in the kinase-RNase domain of IRE1. Molecular cell 38, 291–304 (2010). 20417606 10.1016/j.molcel.2010.04.001 PMC2864793

[b19] Trott O. & Olson A. J. AutoDock Vina: improving the speed and accuracy of docking with a new scoring function, efficient optimization, and multithreading. J Comput Chem 31, 455–461 (2010). 19499576 10.1002/jcc.21334 PMC3041641

[b20] Spiotto M. T. *et al.* Imaging the unfolded protein response in primary tumors reveals microenvironments with metabolic variations that predict tumor growth. Cancer Res 70, 78–88 (2010). 20028872 10.1158/0008-5472.CAN-09-2747 PMC2943832

[b21] Benosman S. *et al.* Interleukin-1 receptor-associated kinase-2 (IRAK2) is a critical mediator of endoplasmic reticulum (ER) stress signaling. PloS one 8, e64256 (2013). 23724040 10.1371/journal.pone.0064256 PMC3665826

[b22] Cross B. C. *et al.* The molecular basis for selective inhibition of unconventional mRNA splicing by an IRE1-binding small molecule. Proc Natl Acad Sci USA 109, E869–878 (2012). 22315414 10.1073/pnas.1115623109 PMC3326519

[b23] Kriss C. L. *et al.* Overexpression of TCL1 activates the endoplasmic reticulum stress response: a novel mechanism of leukemic progression in mice. Blood 120, 1027–1038 (2012). 22692508 10.1182/blood-2011-11-394346 PMC3680046

[b24] Bright M. D., Itzhak D. N., Wardell C. P., Morgan G. J. & Davies F. E. Cleavage of BLOC1S1 mRNA by IRE1 Is Sequence Specific, Temporally Separate from XBP1 Splicing, and Dispensable for Cell Viability under Acute Endoplasmic Reticulum Stress. Molecular and cellular biology 35, 2186–2202 (2015). 25870107 10.1128/MCB.00013-15 PMC4438243

[b25] Pommier Y., Leo E., Zhang H. & Marchand C. DNA topoisomerases and their poisoning by anticancer and antibacterial drugs. Chemistry & biology 17, 421–433 (2010). 20534341 10.1016/j.chembiol.2010.04.012 PMC7316379

[b26] Binaschi M. *et al.* Comparison of DNA cleavage induced by etoposide and doxorubicin in two human small-cell lung cancer lines with different sensitivities to topoisomerase II inhibitors. International journal of cancer. Journal international du cancer 45, 347–352 (1990). 2154411 10.1002/ijc.2910450223

[b27] Tidefelt U., Sundman-Engberg B. & Paul C. Intracellular uptake and cytotoxic effect *in vitro* of doxorubicin and epirubicin in human leukemic and normal hematopoietic cells. Cancer chemotherapy and pharmacology 29, 7–12 (1991). 1742852 10.1007/BF00686328

[b28] Anderson K. C. *et al.* Multiple Myeloma: New Insights and Therapeutic Approaches. Hematology/the Education Program of the American Society of Hematology. American Society of Hematology. Education Program 147–165 (2000). 11701540 10.1182/asheducation-2000.1.147

[b29] Orlowski R. Z. *et al.* Phase 1 trial of the proteasome inhibitor bortezomib and pegylated liposomal doxorubicin in patients with advanced hematologic malignancies. Blood 105, 3058–3065 (2005). 15626743 10.1182/blood-2004-07-2911

[b30] Lu M. *et al.* Prevention of Doxorubicin cardiopathic changes by a benzyl styryl sulfone in mice. Genes & cancer 2, 985–992 (2011). 22701764 10.1177/1947601911436199 PMC3374628

[b31] Hu C. C., Dougan S. K., McGehee A. M., Love J. C. & Ploegh H. L. XBP-1 regulates signal transduction, transcription factors and bone marrow colonization in B cells. The EMBO journal 28, 1624–1636 (2009). 19407814 10.1038/emboj.2009.117 PMC2684024

