# Supplementary material for: Identification of Doxorubicin as an Inhibitor of the IRE1α-XBP1 Axis of the Unfolded Protein Response
Source: Sci Rep. 2016 Sep 16;6:33353. doi: 10.1038/srep33353 (PMC5025885; doi:10.1038/srep33353)
Supplement: Supplementary Information [file srep33353-s1.pdf]

Identification of Doxorubicin as an Inhibitor of the IRE1α-XBP1 Axis of the Unfolded Protein Response

Dadi Jiang, Connor Lynch, Bruno C. Medeiros, Michaela Liedtke, Rakesh Bam, Arvin B. Tam, Zhifen Yang, Muthuraman Alagappan, Parveen Abidi, Quynh Thu Le, Amato J. Giaccia, Nicholas C. Denko, Maho Niwa and Albert C. Koong

Supplementary Figure S1

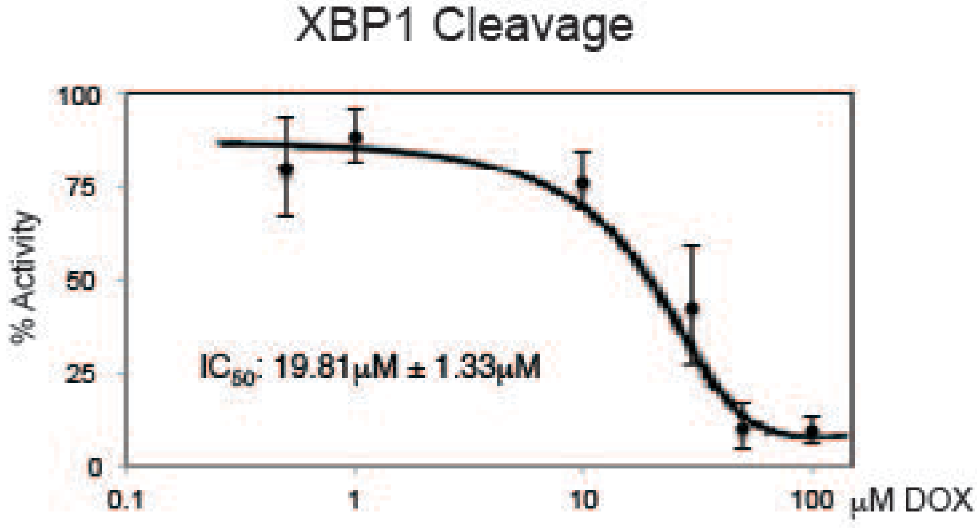

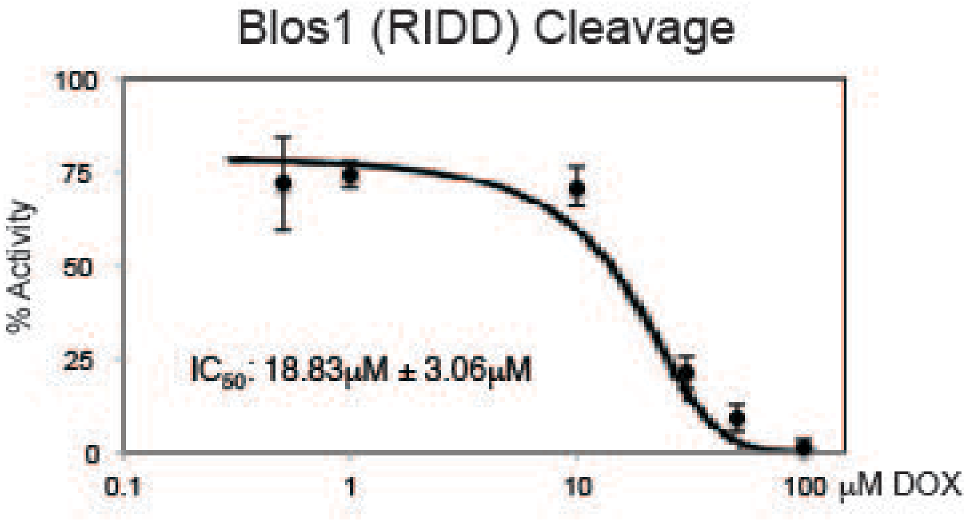

Supplementary Figure S1. Inhibition of XBP1 splicing and RIDD activities of IRE1α by doxorubicin *in vitro*

In vitro RNase reactions with purified recombinant IRE1α and radiolabelled XBP1 or BLOS1 (representing RIDD activity) mRNA were performed in the presence of 0, 0.5, 1, 10, 30, 50, 100 μM doxorubicin. Doxorubicin demonstrated inhibition of IRE1α RNase activity with an IC_{50} calculated to be 19.81 ± 1.33 μM for XBP1 splicing and 18.83 ± 3.06 μM for RIDD.

Supplementary Figure S2

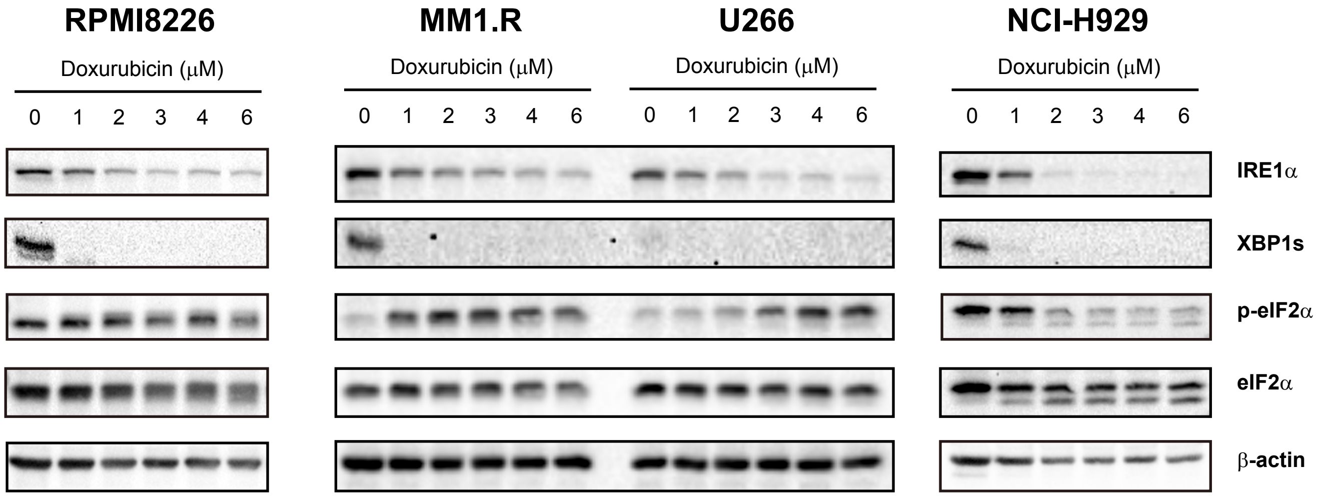

Supplementary Figure S2. The effect of doxorubicin on UPR signaling proteins in MM cell lines
Western blot analysis of UPR signaling proteins in four MM cell lines treated with varying doses of doxorubicin for 14 hours. β-actin was used as a loading control.

Supplementary Figure S3

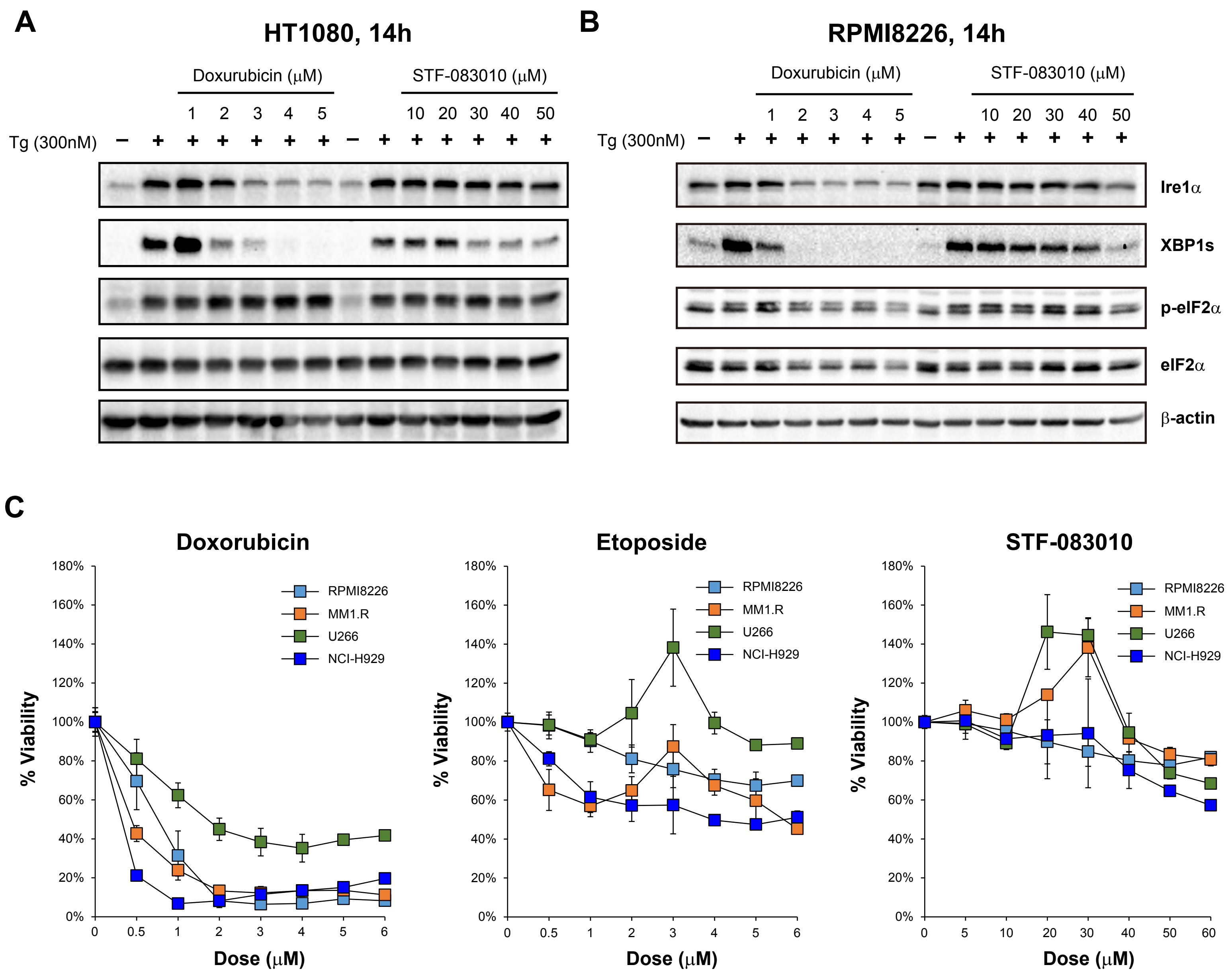

Supplementary Figure S3. Comparison of XBP1s inhibition and cytotoxicity on MM cells between doxorubicin and STF-083010

(A) Western blot analysis of UPR signaling proteins in HT1080 cells treated with 300 nM thapsigargin (Tg) together with varying doses of doxorubicin or STF-083010 for 14 hours. β-actin was used as a loading control. (B) Western blot analysis of UPR signaling proteins in RPMI 8226 cells treated with 300 nM thapsigargin together with varying doses of doxorubicin or STF-083010 for 14 hours. β-actin was used as a loading control. (C) Viability of RPMI 8226, MM1.R, U266 and NCI-H929 MM cells determined by XTT assay after increasing doses of doxorubicin, etoposide, or STF-083010 treatment for 24 hours. Mean ± SD of % viability relative to 0 µM drug (set to 100%) was plotted.

Supplementary Figure S4

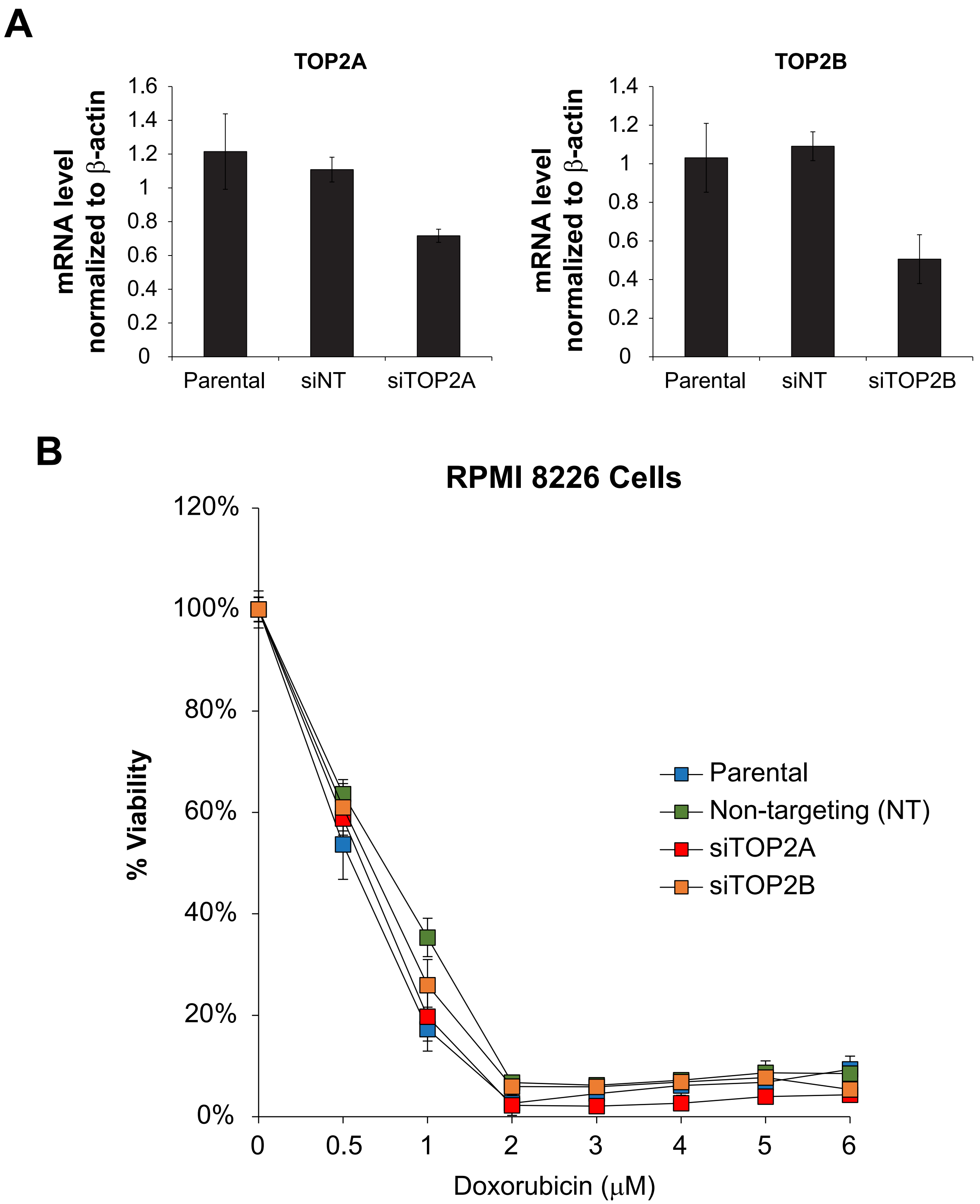

Supplementary Figure S4. Topoisomerase II knockdown doesn't change the sensitivity of MM cells to doxorubicin

(A) Knockdown of TOP2A or TOP2B expression by siRNA was verified by qRT-PCR using primers specific to the isoforms. Results represent average quantities of technical triplicates of the target gene normalized to total β-actin ± c.v. (B) Viability of parental, non-targeting and TOP2A/B-knockdown RPMI 8226 cells determined by XTT assay after increasing doses of doxorubicin treatment for 24 hours. Mean ± SD of % viability relative to 0 μM doxorubicin (set to 100%) was plotted.

Supplementary Figure S5

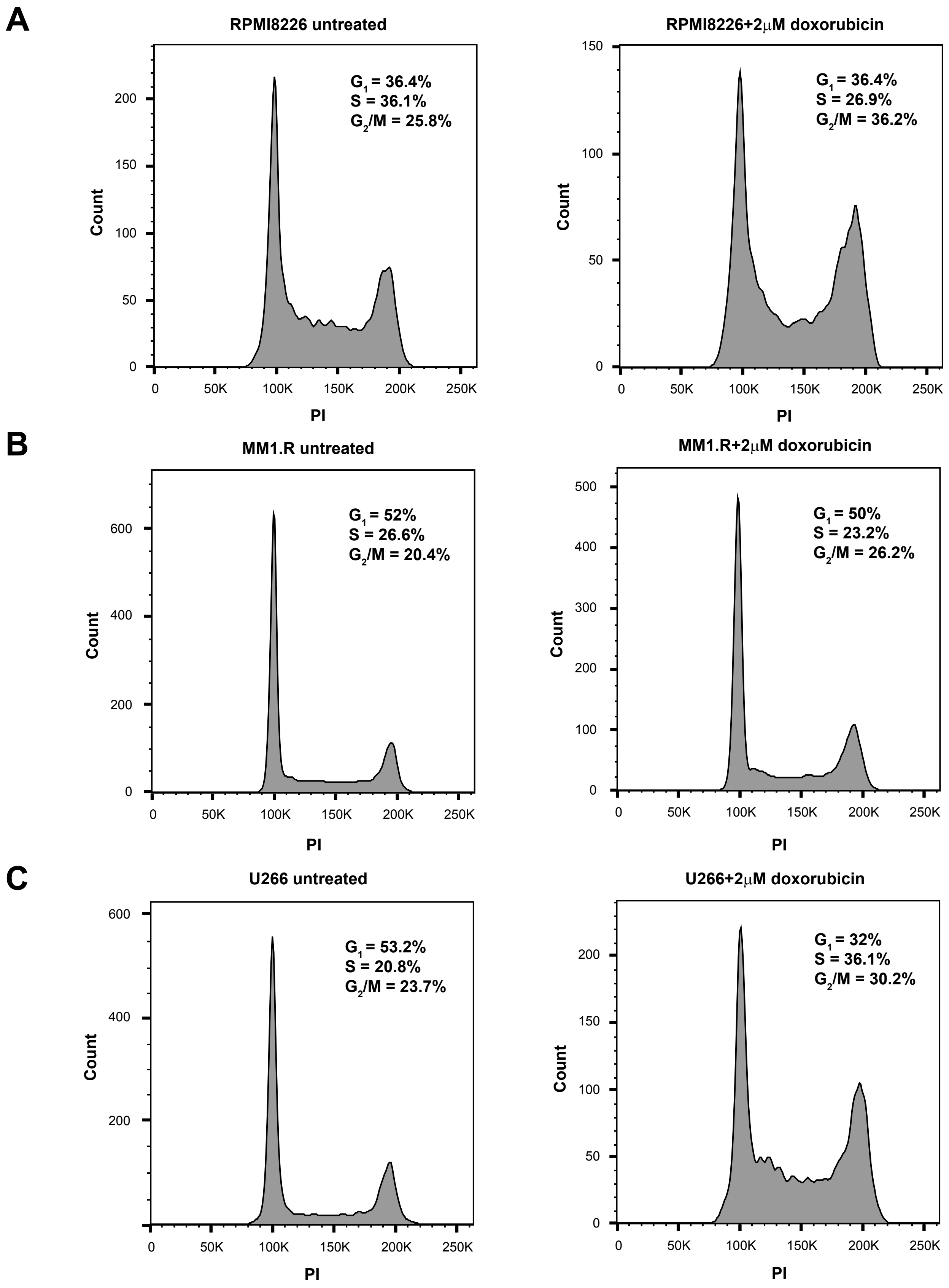

Supplementary Figure S5. Cell cycle analysis of doxorubicin-treated MM cell lines
RPMI 8226 (A), MM1.R (B) and U266 (C) cells were left untreated or treated with 2 μM doxorubicin for 24 hours. Cell cycle distribution was analyzed by propidium iodide (PI) staining and FACS analysis. The proportion of each cell cycle was estimated in FlowJo (v10) and labeled in the corresponding panel.

Supplementary Figure S6

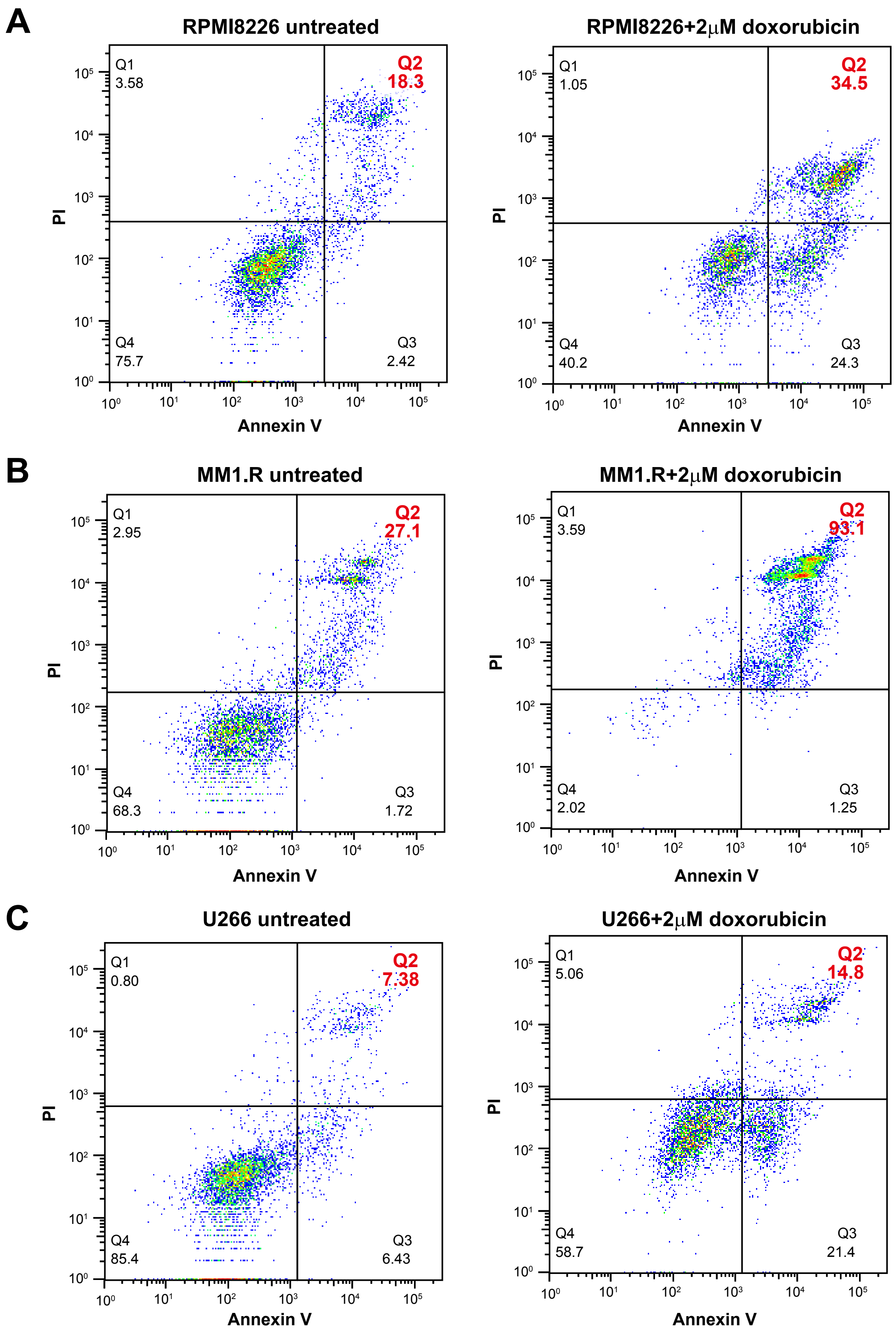

Supplementary Figure S6. Apoptosis analysis of doxorubicin-treated MM cell lines
RPMI 8226 (A), MM1.R (B) and U266 (C) cells were left untreated or treated with 2 μM doxorubicin for 24 hours. Apoptosis was analyzed by Annexin V-PI staining and FACS analysis. The proportion of each quadrant was estimated in FlowJo (v10) and labeled in the corresponding panel.

Supplementary Figure S7

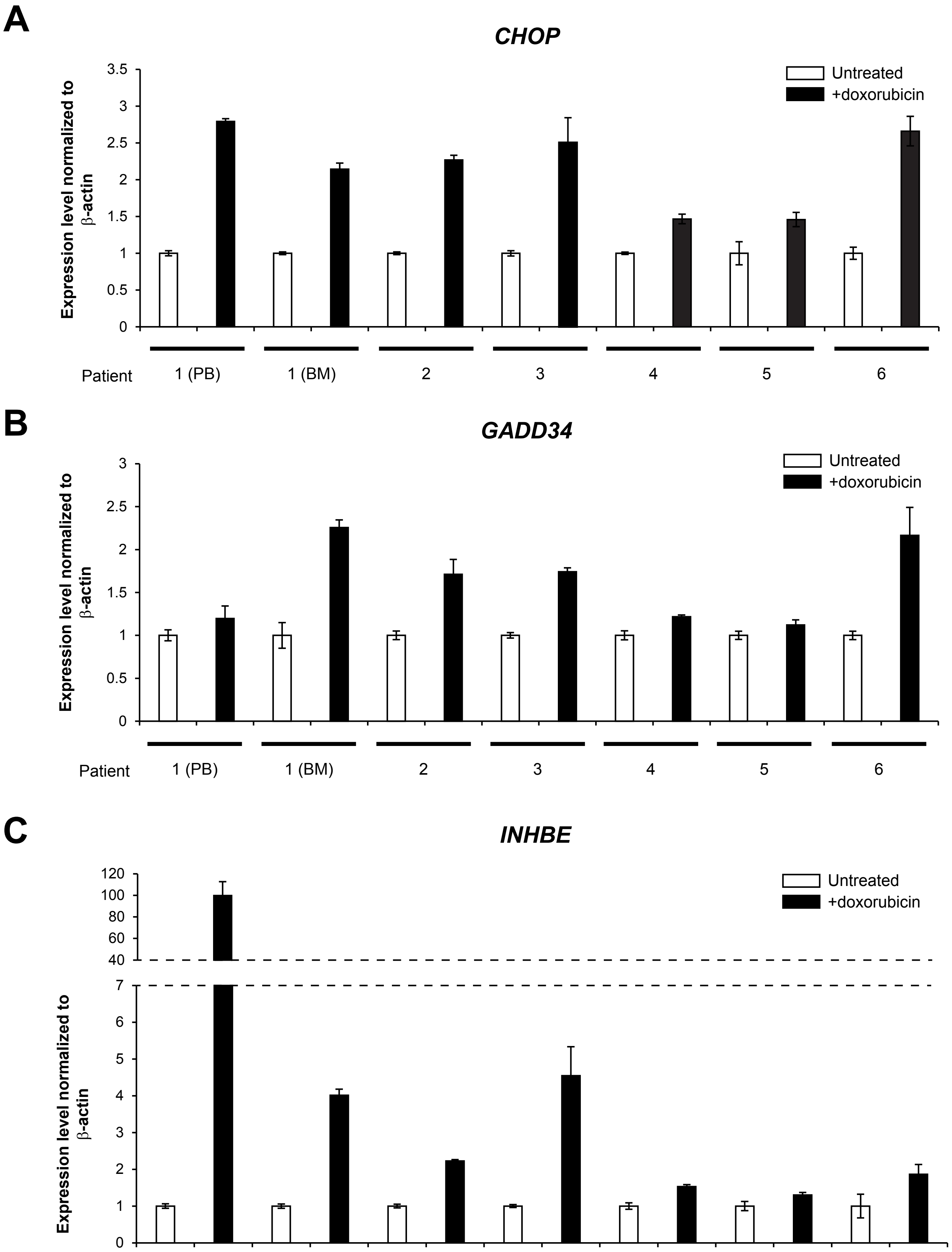

Supplementary Figure S7. Doxorubicin induces the expression of PERK-ATF4 target genes

Doxorubicin induced the expression of (A) *CHOP*, (B) *GADD34* and (C) *INHBE* in $CD138^+$ cells freshly isolated from MM patients undergoing active therapy. $CD138^+$ cells isolated from 6 MM patients were treated with 1 μM doxorubicin for 6 hours then total RNA was extracted and used for qRT-PCR analysis with primers specific for the individual genes. Results represent average quantities of technical triplicates of the target gene normalized to total β-actin ± c.v. Results of $CD138^+$ cells from both peripheral blood (PB) and bone marrow (BM) of patient 1 are shown.
